# Multifaceted analyses reveal carbohydrate metabolism mainly affecting the quality of postharvest bamboo shoots

**DOI:** 10.3389/fpls.2022.1021161

**Published:** 2022-09-21

**Authors:** Zhen Li, Xiurong Xu, Kebin Yang, Chenglei Zhu, Yan Liu, Zhimin Gao

**Affiliations:** ^1^International Centre for Bamboo and Rattan, Institute of Gene Science and Industrialization for Bamboo and Rattan Resources, Beijing, China; ^2^Key Laboratory of National Forestry and Grassland Administration/Beijing for Bamboo and Rattan Science and Technology, Beijing, China; ^3^Zhejiang Academy of Forestry, Hangzhou, China

**Keywords:** *Phyllostachys edulis* shoot, postharvest physiology, carbohydrate metabolism, co-expression network, xylan biosynthesis

## Abstract

Bamboo shoot is one of nutritious vegetables in China. However, the edible quality of fresh bamboo shoots deteriorates easily after harvest. Here, morphological, physiological, transcriptomic and microRNA sequencing analyses were conducted to investigate the postharvest characteristics of moso bamboo (*Phyllostachys edulis*) shoots. Rapid decreases of soluble sugars, structural polysaccharides and hydrolyzed tannins, and increases of lignin and condensed tannins were observed in the postharvest bamboo shoots. Differentially expressed genes (DEGs) and miRNAs with opposite trends were mainly enriched in structural polysaccharide metabolism, starch and sucrose metabolism and glycolysis pathways, which were consistent with the changes of carbohydrates. A co-expression network of carbohydrate metabolism was constructed, which was verified by qPCR and yeast one-hybrid (Y1H) assay. Furthermore, the function of one hub glycosyltransferase gene was validated in *Arabidopsis*, which confirmed that it was involved in xylan biosynthesis. These results are of great significance for revealing the carbohydrate metabolism mechanisms of postharvest bamboo shoots and provide a potential candidate gene for molecular breeding related to xylan in the future.

## Introduction

Bamboo shoots, a kind of immature and expanding culms generated from the nodes of bamboo pseudo-rhizomes, are usually considered as a highly desirable vegetable because of their sweet taste, crisp and crunchy texture and excellent nutrition (Singhal et al., 2013). Carbohydrates in bamboo shoots are abundant and available in multiple forms, with the largest proportion of polysaccharides including starch as an energy source, and cellulose, and hemicellulose as dietary fibers. The dietary fiber of bamboo shoots accounts for 50% of carbohydrates (Wang et al., 2020), playing a critical role in hypolipidemic and anti-obesity activities as well as the taste (Tanabe et al., 2017). However, due to the perishability of bamboo shoots, the shelf life and market value of fresh bamboo shoots have been restricted. The soluble sugar content of bamboo shoots decreased significantly at the beginning of storage, e.g., the starch content of *Fargesia yunnanensis* shoots decreased significantly after 3 days of storage (Yu et al., 2021). Meanwhile, the changes in phenolic content of bamboo shoots led to browning and increased lignin content, along with the phenylalanine ammonia-lyase, and cinnamyl alcohol dehydrogenase activities increased rapidly (Li X. et al., 2019).

Therefore, several studies have focused on storage and food processing technology by both physical and chemical methods. In terms of physical methods, such as cooling, UV-C, gamma radiation and hypobaric atmosphere are often applied to extend the shelf life of bamboo shoots, which prolong the edible time of bamboo shoots by reducing the accumulation of lignin and chlorophyll (Wang et al., 2019; Wen et al., 2019). As for chemical methods, hormone treatment is commonly used to keep vegetable fresh. Exogenous melatonin and diphenyliodonium iodide treatment effectively delay the lignification of bamboo shoot and reduce the rate of yellowing during cold storage (Li C. et al., 2019; Li D. et al., 2019), while brassinolide treatment alleviates chilling injury of bamboo shoots by enhanced enzyme activities related to energy and proline metabolism (Liu et al., 2016). However, even low-temperature (LT) storage cannot stop the lignification process. Moreover, many techniques cannot be applied for ordinary households, especially bamboo shoot farmers living in remote mountainous areas who have difficulty using refrigerators. In view of the mechanisms underlying these physical and chemical methods, it suggests that the regulation of carbohydrate metabolism is important for affecting bamboo shoot edible quality and shelf life. Although similar biological processes occur in other harvested vegetables and fruit, and the effect of carbohydrate metabolism on quality is relatively clear, those in bamboo shoots are still unclear.

Structural polysaccharide metabolism, starch, and sucrose metabolism and glycolysis are the main carbohydrate metabolisms. Structural polysaccharides affected the juiciness and adhesion of cells, and promoted fruit soften or granulation. Propectin and cellulose showed significant increase in postharvest granulated orange, and the genes encoding enzymes responsible for pectin and cellulose, e.g., galacturonosyltransferase (GAUT) and cellulose synthase complex were up-regulated, while that of the gene encoding polygalacturonase responsible for pectin degradation showed significant decrease (Yao et al., 2020). In postharvest kiwifruits and persimmon fruit, the protopectin and cellulose content decreased while water-soluble pectin content increased progressively, accompanied by the increased activities of UDP-xylose 4-epimerase, UDP-Glc dehydrogenase, pectinesterase and cellulase (Tian et al., 2021). Genes encoding sucrose phosphate synthase (SPS), invertase (INV), and α-amylase (AMY) showed significantly differential expression in Powell (*Citrus sinensis*), fresh lotus seeds and kiwifruits during storage (Sun et al., 2021; Tian et al., 2021). Those genes encoding pyruvate kinase (PK) and phosphofructokinase related to glycolysis are highly variable during storage in many kinds of fruit and vegetables, such as sugarbeet roots, lotus seeds, and kiwifruits (Tian et al., 2021). Within sucrose and starch cycle, starch synthesis and degradation are simultaneously, during the preharvest period, taking place with net breakdown thereafter (Schouten et al., 2016). In addition, transcription factors (TFs) like bHLH, MYB, ERF, and LBD play important roles in regulating postharvest physiological changes (Wu et al., 2021).

Transcriptome sequencing is a powerful tool to rapidly obtain information on the expressed fraction of a genome, including the gene expression patterns, and the small ribonucleic acid (RNAs) of the targeted genes. The molecular mechanism of some physiological changes in postharvest bamboo shoots has been preliminarily analyzed, and the pathways of secondary cell walls and hormone biosynthesis are identified (Zhang et al., 2018). It was noticed that genes involved in lignin and hemicellulose biosynthesis were up-regulated and down-regulated, respectively (Li et al., 2018; Zhang et al., 2020). In addition, NAC, bHLH, bZIP, MYB, and WRKY were identified as critical TFs for shoot postharvest senescence, in which PheNAP2 and PheNAP3 were found to promote leaf senescence in *Arabidopsis* (Li X. et al., 2019). The expression of jasmonic acid (JA) biosynthesis related genes was highly consistent with that of lignin precursor biosynthesis genes under low temperature (LT), indicating a LT-lignification or LT-JA-lignification regulatory pathway existed in Lei bamboo shoots (Hou et al., 2022). However, the molecular mechanism affecting edible quality associated with carbohydrates in postharvest shoots is still unclear. In this study, the characteristics related to the main carbohydrate metabolism of postharvest moso bamboo shoots were revealed by integrated analyses of anatomical observation, chemical component and enzyme activity determination, transcriptomic and microRNA sequencing, which provided new insight into the modulation affecting the quality of postharvest moso bamboo shoots.

## Materials and methods

### Materials, treatment and ribonucleic acid extraction

**FIGURE 1:**
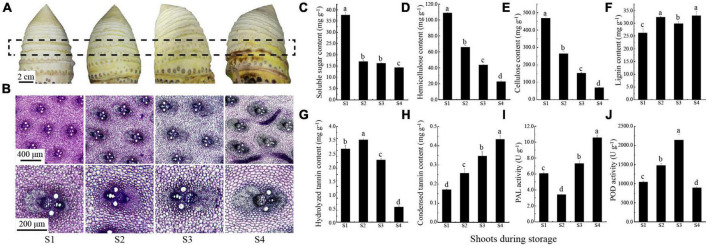
Changes of postharvest moso bamboo shoots. **(A)** Morphological changes of shoots after postharvest 0, 3, 6, and 12 d (S1: 0 d; S2: 3 d; S3: 6 d; S4: 12 d). **(B)** Staining intensity of vascular bundle transverse section of the boxed area in **(A)**. **(C–H)** The content of soluble sugar **(C)**, hemicellulose **(D)**, cellulose **(E)**, lignin **(F)**, hydrolyzed tannins **(G)** and condensed tannins **(H)**, the activities of PAL **(I)** and POD **(J)**. Lowercase letters a, b, c, and d indicate significant differences (*P* < 0.05).

The moso bamboo (*Phyllostachys edulis*) shoots of 15 cm ∼ 20 cm in length and 5 cm ∼ 8 cm in basal diameter were harvested from a wild bamboo forest (28°45′58″N, 115°45′39″E, 399.0 m above sea level) in Nanchang, Jiangxi province, China, in December 2019. The shoots were stored under room temperature conditions (22 ± 2°C, RH = 75%) wrapped in paper bags. Middle part of the shoots in Figure 1A were collected at different storage time (S1, S2, S3, and S4 represented bamboo shoots stored for 0, 3, 6, and 12 d), one part of samples was immediately frozen in liquid nitrogen and then stored at −80°C, another part of samples was fixed with FAA and stored at 4°C until use, respectively. Three biological replicates were set for each time point. The total RNA and small RNA from 12 samples was isolated using the TRIzol and miRcute plant miRNA extraction and isolation reagent (item numbers DP504, TIANGEN Biotech Co., Ltd., Beijing), respectively.

### Histological staining, determination of compound content and enzyme activity

The shoot samples were stained with 5% (v/v) of toluidine blue (TBO), as described in the reference (Yang et al., 2021). The content of soluble sugar, cellulose, hemicellulose, lignin, hydrolyzed tannins, and condensed tannins, and the enzyme activity of peroxidase (POD) and L-phenylalanine ammonia lyase (PAL) were determined with detection kits (item numbers G0501, G0519, G0527, G0708, G0119, G0120, G0114, and G0107) of Grace Biotechnology Co., Ltd., JiangSu. The content of xylan was determined by immunofluorescence with detection kits (item numbers S0169O) of KETE Biotechnology Co., Ltd., Jiangsu. Three replicates were performed for each determination.

### Transcriptome and small ribonucleic acid sequencing

A total 12 transcriptome and 12 small RNA libraries were generated using NEBNext UltraTM RNA Library Prep Kit for Illumina (NEB, USA) and NEBNext Multiplex Small RNA Library Prep Set for Illumina (NEB, USA) following manufacturer’s recommendations, respectively. Illumina platform was used to sequence 12 high quality RNA libraries at Biomarker Technologies Corporation (Beijing, China). Low quality reads, reads with adaptors and reads with unknown bases were removed from the data sets to obtain the clean reads, which were then mapped to the reference genome of moso bamboo, followed by unigene expression level calculation with fragments per kilobase of transcript per million fragments mapped (FPKM) in each sample.

Differential expression analysis was performed using DESeq2 (Love et al., 2014), and genes with Fold Change (FC) ≥ 2 and False Discovery Rate (FDR) < 0.01 were assigned as differentially expressed (Tarazona et al., 2011). The putative functions of the differentially expressed genes (DEGs) and target genes were determined according to Non-redundant protein (NR) database. The cluster classification of DEGs based on expression of their transcriptome profiles were performed using OmicShare Tools^1^ with default parameters. The Gene Ontology (GO) enrichment analysis was carried out using R-based GOseq package to identify the over-represented functional categories, and Kyoto Encyclopedia of Genes and Genomes (KEGG) enrichment analysis was implemented by KOBAS 3.0 (Xie et al., 2011). For co-expression network analysis, the weighted gene co-expression network analysis (WGCNA) package was used. Correlation analysis of gene expression was performed using programs in the BMKCloud.^2^ Based on the results of WGCNA, the genes in the module with the highest carbohydrate correlation were selected to construct the co-expression network. DEGs related to structural polysaccharide metabolism, starch, and sucrose metabolism and glycolysis were screened from this module by gene function annotation. The upstream sequences of these DEGs were further analyzed to identify potential regulatory TFs in turquoise module combining with biological information in PlantTFDB databases.^3^ The small RNA sequencing data from this study and psRNATarget database^4^ were used to identify potential regulatory miRNAs targeting these DEGs.

For Small RNA data analysis, clean reads of small RNA were obtained by removing reads containing adapter and ploy-N besides low-quality reads using Trimmomatic v0.35. After screening against reads shorter than 18 nt and longer than 35 nt, rRNA, tRNA, snoRNA, and repeat sequences, the redundant reads were processed into unique sequences with associated read counts for miRNA prediction by comparing with genome and known miRNAs from miRBase using Bowtie alignment tool v1.1.2 with two mismatches. Randfold tools software was used for novel miRNA secondary structure prediction. Subsequently, the mRNA targets of the identified miRNAs were predicted using psRNATarget server with default parameters. The annotation of these target genes refers to the method of DEG annotation in 2.3. The miRNAs expression determination and differentially expressed miRNAs (DEMs) analysis refers to the method of transcriptome analysis.

### Validation of differentially expressed genes and differentially expressed miRNAs

Primer Premier 5.0 software was used to design the specific primers based on the candidate genes and miRNA sequences (Supplementary Table 1A). The qPCR reactions were performed with the SYBR Green Master Mix in a qTOWER 2.2 system (Analytik Jena, Germany) with four technical replicates, using *PeTIP41* (Fan et al., 2013) and *U6* snRNA (Yang et al., 2021) as the endogenous controls for the genes and miRNAs, respectively. The qPCR conditions were as follows: 95°C for 6 min, followed by 40 cycles at 95°C for 15 s and 60°C for 15 s. The expression levels were calculated using the 2^–ΔΔCT^ method (Livak and Schmittgen, 2001).

### Yeast one-hybrid assay

The 5′ UTR and promoter sequences of PH02Gene04386 (a glycosyltransferase 43 gene) were segmented into four sequences with different lengths, including 5′ UTR sequence of 600 and 1,119 bp upstream of cDNA namely 5′ UTR-600 and 5′ UTR-1119, promoter sequence of 600 and 1,200 bp upstream of mRNA namely pro-600 and pro-1200, respectively. The coding sequence (CDS) of two *PeMYB*s (PH02Gene12213 and PH02Gene31429) were recombined into the pGADT7-Rec2 vector. Six SMRE element (SMRE2 ∼ SMRE7) regions and four 5′ UTR/promoter sequences of PH02Gene04386 were inserted into the pHIS2 vector, respectively. Two recombinant vectors were co-transformed into the Y187 strain. The transformants were screened on Leu-, Trp-, and His-deficient medium supplemented with 20 mM of 3-amino-1,2,4-triazole for 3 d, using cotransformants containing p53:His2 with pGADT7:53 and p53:His2 with pGADT7:PeMYBs as the positive control and negative control, respectively. The primers used in yeast one-hybrid (Y1H) assay were listed in Supplementary Table 1B.

### Functional validation of PH02Gene04386

The CDS of PH02Gene04386 was isolated and cloned into the expression vector of pSuper1300. The transgenic plants overexpressing (OE) PH02Gene04386 were obtained through ectopic expression in *Arabidopsis* (Col-0) plants using the floral dip method, which were further verified by PCR and qPCR. Root length of transgenic and wild-type (WT) *Arabidopsis* seedlings was counted after 7 days growing on Murashige and Skoog medium with 50 mM xylose as the carbon source. The main inflorescence stem (1 ∼ 2 cm aboveground) of both transgenic and WT *Arabidopsis* plants of 6 weeks were used for histological staining with 5% (v/v) of TBO, xylan immunofluorescence localization with LM10 (Keppler and Showalter, 2010) and xylan content analysis, respectively. The fluorescence signals of FITC were recorded with a Zeiss LSM 980 confocal microscope. Images were taken with an Olympus CX31 light microscope (Tokyo, Japan).

### Statistical analysis

A standard *t*-test is used to determine statistical significance with a 95% confidence interval. In all figures, data are represented as the mean ± standard error (SE).

## Results

### Changes of morphology, physiology, and enzyme activities in moso bamboo shoots

During storage at room temperature, the surface of bamboo shoots changed significantly, and gradually browned with time (Figure 1A). Meanwhile, the degree of staining intensity of lignin was deepening and the polysaccharides was lightening in vascular bundle (Figure 1B), which indicated a potential change in carbohydrate and phenols content. Since the carbohydrates and phenols are considered to be important factors affecting postharvest edible quality, the content of these substances such as soluble sugar, hemicellulose, cellulose, lignin, and procyanidins in postharvest moso bamboo shoots are measured. The results showed that the continuous decreases were observed in soluble sugar from 37.75 mg g^–1^ (S1) to 14.36 mg g^–1^ (S4), hemicellulose from 108.92 mg g^–1^ (S1) to 22.54 mg g^–1^ (S4) and cellulose from 468.68 mg g^–1^ (S1) to 69.99 mg g^–1^ (S4) (Figures 1C–E). In contrast, the lignin content showed an increase from 26.23 mg g^–1^ (S1) to 32.91 mg g^–1^ (S4) (Figure 1F). The content of hydrolyzed tannin increased slightly from 3.07 mg g^–1^ (S1) to 3.45 mg g^–1^ (S2) and then significantly decreased to 0.63 mg g^–1^ (S4) (Figure 1G). Meanwhile, the content of condensed tannin (procyanidin) increased continuously, and doubled that of S1 (0.17 mg g^–1^) at the end (S4, 0.43 mg g^–1^) (Figure 1H). The activity of PAL was first decreased from 6.08 U g^–1^ (S1) to 3.41 U g^–1^ (S2), and then increased significantly to 10.55 U g^–1^ (S4) (Figure 1I). The activity of POD peaked at 2140.90 U g^–1^ (S3), but decreased significantly to 897.27 U g^–1^ at the end (S4) (Figure 1J). These results were in agreement with our current understanding of taste and flavor decline in postharvest moso bamboo shoots.

### Analysis of transcriptome sequencing and differentially expressed genes in moso bamboo shoots

To further understand the molecular mechanisms of above changes, 12 transcriptome libraries were constructed, with 325,040,670 clean reads generated. The clean data were mapped to the reference genome of moso bamboo with the mapping ratio varying from 96.16 to 96.66% (Supplementary Table 2A). Meanwhile, 7,458 unigenes were identified, among which 5,061 were functionally annotated (Supplementary Table 2B). A total 12,553 DEGs were identified by comparing the samples (Supplementary Figures 1A,C). Annotation analysis showed that there were 1,046 DEGs encoding TFs belonging to 44 families, in which MYB (112), bHLH (91), AP2/ERF (80), WRKY (79), and NAC (70) were the most over-represented TF families (Supplementary Figure 1B).

**FIGURE 2:**
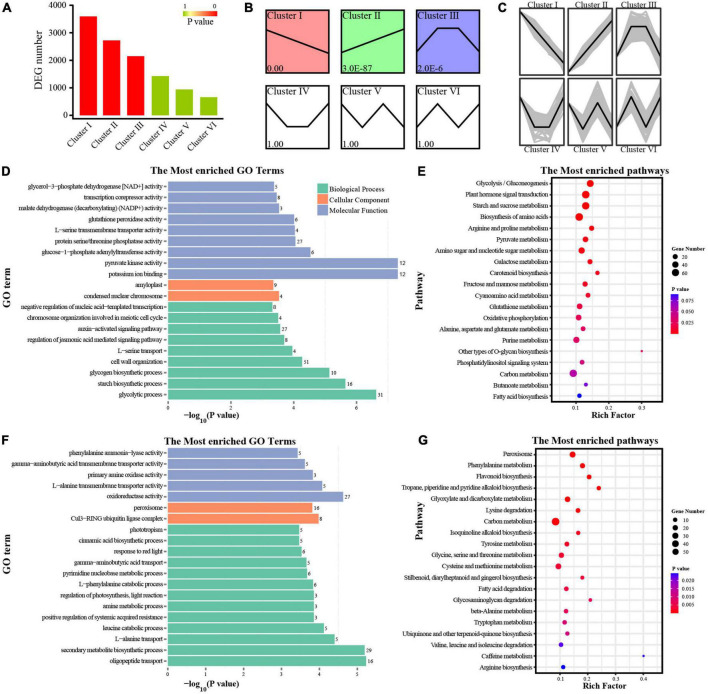
Function enrichment analysis of DEGs. **(A)** DEG number in different clusters. **(B)** Different clusters based on *P*-values. **(C)** DEGs with similar expression trends in a specific cluster. **(D–G)** GO and KEGG analyses of DEGs in cluster I **(D,E)**, cluster II **(F,G)**. Rich Factor represents the value of enrichment factor which is the quotient of foreground value (the number of DEGs), and the larger the value, the more significant enrichment; Coloring indicates *P*-value with higher in red and lower in blue, and the lower *P*-value, the more significantly enriched. Point size indicates the number of DEGs.

We performed expression analysis of the DEGs, and grouped genes according to the similar expression of their transcriptome profiles. A total of six clusters were identified (Figures 2A–C), and most of the DEGs belonged to two kinds of clusters: down-regulated (cluster I, 3,596 DEGs) and up-regulated (cluster II, 2,723 DEGs). In cluster I, GO and KEGG analyses showed that the DEGs were enriched in carbohydrate metabolism (Figures 2D,E). Different from cluster I, DEGs in cluster II were related to secondary metabolism (Figures 2F,G). The results of GO and KEGG analyses in cluster III showed that DEGs were enriched in a variety of pathways, including photosynthesis, carbohydrate and secondary metabolism (Supplementary Figures 2A,B). Considering the functional terms in combination with morphological characteristics, we proposed that the decrease of soluble sugar and structural polysaccharides along with the increase of lignin and procyanidins may be due to the expression changes of DEGs involved in carbohydrate metabolism and secondary metabolism. Among them, carbohydrate-related DEGs were the most abundant, which was consistent with the significant changes in carbohydrate content.

### Expression patterns of carbohydrate metabolism related differentially expressed genes in moso bamboo shoots

**FIGURE 3:**
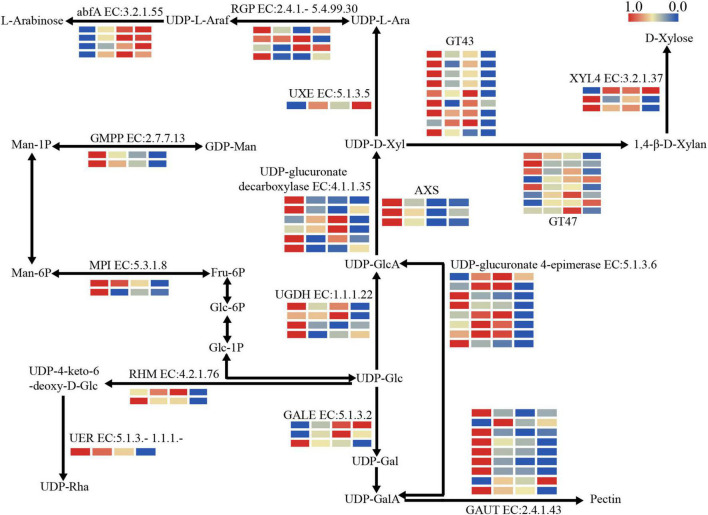
The expression patterns of DEGs involved in structural polysaccharide metabolism. abfA, alpha-L-arabinofuranosidase; AXS, UDP-apiose/xylose synthase; GALE, UDP-glucose 4-epimerase; GAUT, α-1,4-galacturonosyltransferase; GT43, glycosyltransferase 43; GT47, glycosyltransferase 47; RGP, eversibly glycosylated polypeptide; RHM, UDP-glucose 4,6-dehydratase; UER, 3,5-epimerase/4-reductase; UGDH, UDP-glucose 6-dehydrogenase; UXE, UDP-arabinose 4-epimerase; XYL4, xylan 1,4-beta-xylosidase. The color bar indicates log_2_-based fragments per kilobase per million (FPKM) with higher in red and lower in blue.

To investigate the changes in carbohydrate metabolism, the expression patterns of DEGs in three key metabolic pathways were further analyzed. The result showed that DEGs were remarkably enriched in the pathway related to nucleotide sugar metabolism. Most of the DEGs were down-regulated, especially those involved in pectin and xylan biosynthesis, while the DEGs involved in arabinose biosynthesis were up-regulated during storage (Figure 3). Three DEGs encoding UDP-apiose/xylose synthase (AXS) and four DEGs encoding UDP-glucose 6-dehydrogenase (UGDH, EC:1.1.1.22) enriched in nucleotide sugar metabolism pathway were identified, with all three AXS DEGs and three of four UGDH DEGs down-regulated significantly. In addition, one DEG encoding UDP-arabinose 4-epimerase (UXE, EC:5.1.3.5) involved in arabinose biosynthesis was identified and its expression increased with storage time.

Further, the DEGs related to nucleotide sugar polymerization were identified, such as those encoding glycosyltransferase family 43 (GT43, EC:2.4.2.-) and glycosyltransferase family 47 (GT47, EC:2.4.2.-) associated with xylan biosynthesis. Most of nine GT43 DEGs were down-regulated significantly during storage, while the expression trends were significantly different among GT47 DEGs, in which the expression of PH02Gene05811 was significantly down-regulated and PH02Gene12063 was first up-regulated and then down-regulated. In addition, the DEGs involved in pectin synthesis were also identified. For example, DEGs encoding GAUT (EC:2.4.1.43) changed significantly during storage, with most of them showing a significant decrease at S2 immediately.

In starch and sucrose metabolic pathways, biosynthesis-related DEGs were down-regulated significantly at S2, e.g., three DEGs encoding starch synthase (glgA, EC:2.4.1.21) and four DEGs encoding SPS (EC:2.4.1.14), while hydrolysis-related DEGs were relatively up-regulated at S2 and S3 (Supplementary Figure 3), e.g., eight DEGs encoding amylase (AMY, EC:3.2.1.1, BMY, EC:3.2.1.2) and six DEGs encoding fructosidase (INV, EC:3.2.1.26). As for Glycolysis, the DEGs encoding enzymes involved in irreversible reactions were down-regulated rapidly at S2, e.g., hexokinase (HK, EC:2.7.1.1) and PK (EC:2.7.1.40) DEGs. However, there were some exceptions, e.g., three DEGs encoding fructose-bisphosphate aldolase (ALDO, EC:4.1.2.13), which were first up-regulated and reached a peak at S3, and then down-regulated (Supplementary Figure 4).

### Expression profiles and targets of small ribonucleic acids in moso bamboo shoots

A total 363.6 million of 418.9 million reads were generated after quality control (Supplementary Table 3A). The length distribution of the unique small RNA reads indicated that 24 nt were the most abundant (36.6%) (Supplementary Table 3B). Further, 47 unique conserved miRNAs and 415 novel miRNAs were identified, with lengths varied from 19 to 25 nt (Supplementary Figures 5A,B), which agreed with many other RNA-Seq experiments (Chen et al., 2020; Li et al., 2021). Of these miRNAs, 451 pre-miRNAs corresponding to 462 mature miRNAs were identified, which were grouped into 93 families (Supplementary Figure 5C and Supplementary Table 3C). The majority of mature miRNAs were characterized by a 5′-uridine residue and the proportion of A and U bases exceed 50%, which was consistent with the characteristics of plant miRNAs attributed to DCL1 cleavage and AGO1 association (Rajagopalan et al., 2006).

**FIGURE 4:**
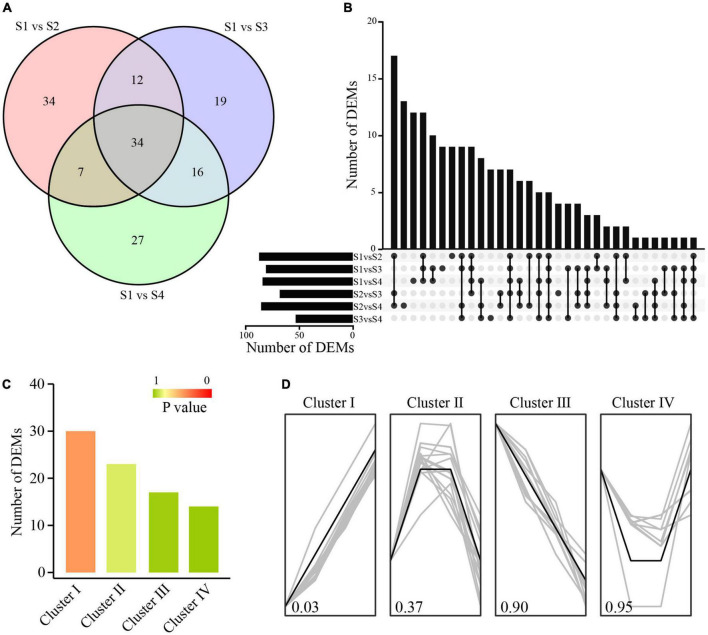
Identification and cluster analysis of DEMs. **(A)** Venn diagram of DEMs. **(B)** Number of DEMs between different comparison groups. **(C)** DEM number in different clusters. **(D)** DEMs with similar expression trends in a specific cluster.

The majority of the identified miRNAs were expressed in more than one sample. In total, 182 miRNAs showed differential expression patterns across different combinations, including 26 known miRNAs and 156 novel miRNAs. There were 34 miRNAs with significant expression changes in moso bamboo shoots at S2, S3, and S4 compared with those at S1 (Figures 4A,B). We merged the DEMs with similar expression profiles to perform cluster analysis. A total of 84 DEMs were clustered into four major clusters with *P*-value less than 0.05 (Figure 4C). The DEMs in cluster I were up-regulated and those in cluster III were down-regulated, while those in cluster II were up-regulated firstly and then down-regulated during storage (Figure 4D). The opposite expression trends between DEMs and DEGs suggested that they might have a potential negative regulatory relationship.

In order to estimate the biological function of these miRNAs, their target genes were identified. A total of 13,748 miRNA-target pairs were identified by TargetFinder and psRNATarget, consisting of 424 miRNAs and 6,024 genes. There were 998 target genes annotated to 125 KEGG pathways. Carbohydrate metabolism and terpenoid and polyketide metabolism were dominant pathways (Supplementary Figure 6), which indicated that transcriptional regulation of carbohydrate metabolism might have important effects on the physiological and biochemical changes in postharvest moso bamboo shoots.

### Co-expression network of differentially expressed genes and differentially expressed miRNAs involved in carbohydrate metabolism of moso bamboo shoots

**FIGURE 5:**
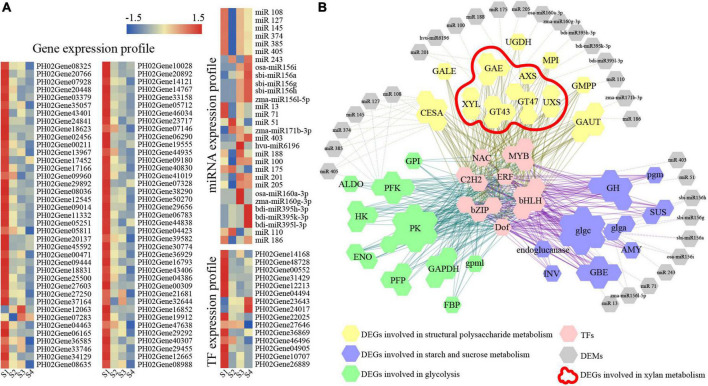
Analysis of DEGs and DEMs involved in carbohydrate metabolism of postharvest moso bamboo shoots. **(A)** Expression patterns of DEGs and DEMs. **(B)** Visualization of co-expression relationships between DEGs and DEMs. The color bar indicates log_2_-based fragments per kilobase per million (FPKM) with higher in red and lower in blue.

**FIGURE 6:**
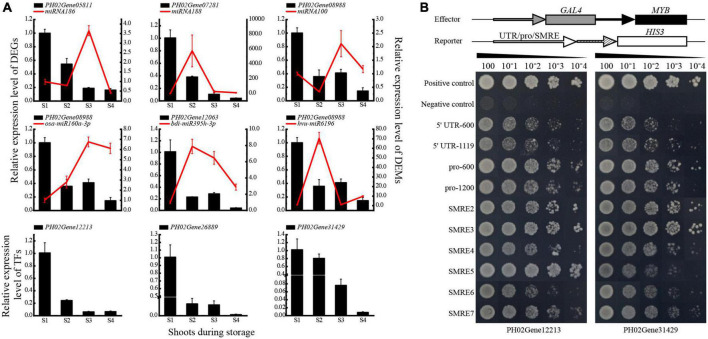
Validation of the genetic elements in the network of carbohydrate metabolism. **(A)** qPCR analysis of DEGs and DEMs. **(B)** Regulatory relationship analysis between MYBs (PH02Gene12213 and PH02Gene31429) and PH02Gene04386 by yeast one-hybrid assay. The red solid lines represent the miRNAs, the black blocks represent the structure genes targeted by miRNAs and MYBs. S1, S2, S3, and S4 represent bamboo shoots stored for 0, 3, 6, and 12 d.

The co-expression network was comprehensively investigated using a WGCNA to predict the role of genes involved in carbohydrate metabolism. A total 4,048 genes with eigengene connectivity (kEM) value more than 0.7 were clustered into four major distinct modules (Supplementary Figure 7A). The turquoise module had a high correlation with sugar (0.99), cellulose (0.96), hemicellulose (0.96), and xylan (0.82), indicating that the genes in this module may be involved in carbohydrate metabolism of postharvest moso bamboo shoots. The correlation between the module and the compounds and expression trends of DEGs were shown in Supplementary Figures 7B,C. The expression of genes and their miRNA pairs in the turquoise module was further analyzed (Figure 5A). Combining the analysis of biological process, motif information, WGCNA, and coherent miRNA-target pairs, a network containing 15 TFs, 78 genes and 30 miRNAs was established, which supported that these TFs and miRNAs were significantly coupled with multiple key enzyme genes related to structural polysaccharides metabolism, starch and sucrose metabolism and glycolysis (Figure 6B). The gene families involved in xylan metabolism were the most numerous, including GT43, GT47, AXS, XYL, and UXS families.

### Validation of the regulatory relationships between genetic elements involved in carbohydrate metabolism

To validate the regulatory network, qPCR and Y1H assays were conducted. Opposite expression trends of miRNA and mRNA in each pair were observed, which was similar to the results of the high-throughput sequencing. Most miRNAs were up-regulated significantly, while their target genes were down-regulated significantly at S2 or S3 (Figure 6A). Additionally, the qPCR results of key TFs, such as two MYBs of PH02Gene12213 and PH02Gene31429, showed similar expression patterns to their target genes, with trends decreasing continuously along with storage time. These results indicated that the enzyme genes might be regulated positively by the TFs and negatively by the miRNAs.

To further validate the regulatory relationship between TFs and genes, the enriched xylan biosynthesis-related gene PH02Gene04386, which was predicted to be a target of MYBs (PH02Gene12213 and PH02Gene31429), was selected for validation of Y1H assay (Figure 6B). The results showed that the MYBs could bind to the 5′ UTR, promoter sequences and SMRE elements of PH02Gene04386. The yeast transformants harboring pro-600 grew best, followed by the transformants harboring pro-1200 and triple SMRE elements. In contrast, the negative control could not grow on the nutritional screening medium. These results indicated that PH02Gene12213 and PH02Gene31429 could bind to the promoter of PH02Gene04386 and might further regulate its expression.

**FIGURE 7:**
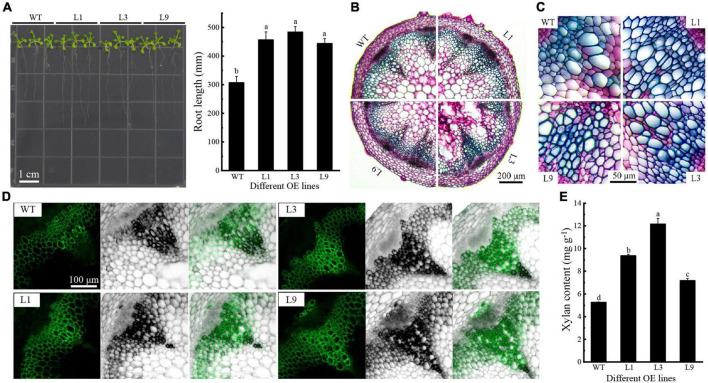
The function analysis of PH02Gene04386 overexpressed in *Arabidopsis*. **(A)** Root length of *Arabidopsis* growing on xylose medium. **(B)** Histochemical staining of cross section of inflorescence stems. **(C)** Vascular bundle in cross section of inflorescence stems in **(B)**. **(D)** Xylan immunofluorescence localization in inflorescence stems. **(E)** Xylan contents in inflorescence stems. Lowercase letters a, b, c, and d indicate significant differences (*P* < 0.05).

PH02Gene04386 was a hub gene in the regulatory network and homologous gene of IRX9, which contributed to the xylan biosynthesis in *Arabidopsis*. The function of PH02Gene04386 was investigated in *Arabidopsis*, and three OE lines (L1, L3, and L9) were used for further analysis (Supplementary Figure 8). The results showed that the primary root length of OE plants was significantly greater than that of WT on 50 mM xylose medium (*P* < 0.05) (Figure 7A). The staining intensity was higher and the vascular bundle cell wall was more thickness in OE flower stems than those of WT (Figures 7B,C). To verify the effect of PH02Gene04386 on the xylan content and distribution in stems of *Arabidopsis* inflorescence, immunofluorescence localization and xylan content analysis were performed using OE and WT plants. The results showed that the fluorescence intensity was higher and the distribution range of xylan antibody (LM10) was broader in OE plants than in WT (Figure 7D). The chemical assay also supported the results that the xylan content was higher in OE plants than in WT (Figure 7E), indicating that overexpression of PH02Gene04386 could promote xylan biosynthesis in *Arabidopsis*. These results supported that PH02Gene04386 was the hub gene in the regulatory network involved in xylan biosynthesis.

## Discussion

### Physiological changes affected quality of postharvest moso bamboo shoots

The edible quality decline rapidly during storage, limiting the spread for fresh bamboo shoots in the market. The shoots of moso bamboo and *P*. *prominens* showed significant lignification during storage, with lower accumulation rate of lignin and cellulose in moso bamboo shoots (Zhang et al., 2020). PAL and POD catalyze the oxidation of polyphenols, which is presumed to be the cause of severe lignification and browning in *P*. *prominens* shoots (Nguyen et al., 2003; Roura et al., 2008). In present study, the degree of browning of moso bamboo shoots is lower than the results in the above study (Figure 1A), probably due to the retention of culm sheaths during storage, thus indicating that storage with culm sheaths is beneficial for preservation of moso bamboo shoots.

The consumption of carbohydrate was an important factor to reduce the edible quality of bamboo shoots. The soluble sugar and hemicellulose contents of the moso bamboo shoots in this study decreased significantly at S2 and continued to decrease during storage (Figures 1C,D), which was similar to the variation of non-structural sugar and hemicellulose in Lei bamboo and green bamboo shoots (Yu et al., 2021). However, the variation of cellulose content was different (Figure 1E; Li et al., 2018; Zhang et al., 2020). It might be explained by the different storage methods and bamboo shoot treatments, by which refrigeration was generally used in the former studies for the shoots with culm sheath removal and bagging in slices, while the shoots with culm sheaths at room temperature used in this study. These results are value for reasonable storage and transportation of bamboo shoots.

### Expression changes of genes affected carbohydrate metabolism

The physiological changes of bamboo shoots during storage depend on the spatial and temporal expression of the relevant genes. In this study, we tried to interpret the molecular mechanism of the physiological changes in moso bamboo shoots based on the expression changes of DEGs. Carbohydrate-related DEGs were the most enriched (Figure 2D), which were closely related to the significant changes of carbohydrate content in moso bamboo shoots. Therefore, it could infer that the carbohydrate-related DEGs had important effect on the edible quality of moso bamboo shoots. Exogenous fructose treatment on Lei bamboo shoots could effectively reduce the lignification and prolong the shelf life by regulating the enzymes involved in starch and sucrose metabolism (Zhou et al., 2020). A large number of DEGs enriched in carbohydrate metabolism such as starch, cellulose and pectin were found in blueberry and African Pride (*Annona cherimola* × *A. squamosa*) during storage (Chen et al., 2019; Cárcamo de la Concepción et al., 2021). In the similar way, the changing pattern of the synthesis-related DEGs in non-structural sugar metabolism was characterized by a decreasing expression and those hydrolysis-related DEGs had an increasing expression along with the physiological changes in this study.

In addition, a large number of DEGs involved in structural polysaccharide metabolism were also identified, among which DEGs involved in the biosynthesis of mannose, xylan and pectin were significantly down-regulated (Figure 3), while those involved in the biosynthesis of arabinoxylans were either up-regulated or first up-regulated and then down-regulated. Studies have shown that the arabinose side chain of xylan binding to the ferulic acid of lignin could increase the stability of the cell wall, and the added xylan acts as a scaffold for lignin dehydrogenation polymer deposition in polysaccharides matrix (Li et al., 2015). This might explain the up-regulation of arabinose biosynthesis related DEGs in this study.

### Carbohydrate metabolism of moso bamboo shoots involved a complex regulatory network

TFs and miRNAs affect carbohydrate metabolism through transcriptional regulation. GhMYB212 regulated the length of fibers and the accumulation of sucrose simultaneously by controlling the expression of a sucrose transporter gene *GhSWEET12* (Sun et al., 2019). Two MYBs of PH02Gene12213 and PH02Gene31429 were suggested to be involved in the biosynthesis of lignin, xylan and cellulose, and lignin deposition, respectively (Yang et al., 2019), both of which had potential regulatory relationships with xylan biosynthesis related DEGs like *GT43* and *GT47* by bioinformatics and Y1H assay (Figures 5B, 6B). A number of miRNAs involved in carbohydrate metabolism were also identified in this study, such as *miRNA167*s and *miRNA160*s (Figure 5B), which had been confirmed to have regulatory roles in starch and sucrose metabolism and structural polysaccharide metabolism in cotton and Acacia (*Acacia auriculiformis* and *A. mangium*) (Wong et al., 2011; Wang et al., 2017). In addition, there were several novel miRNAs in the regulatory network, such as *miRNA100* and *miRNA175*, which provided important information for further investigating the function of miRNAs in polysaccharide metabolism.

**FIGURE 8:**
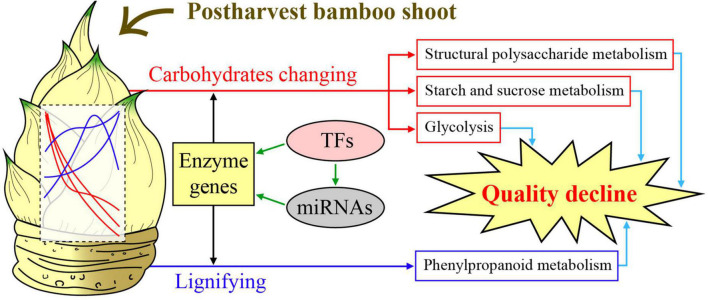
A model to elucidate the regulatory mechanism involved in carbohydrate metabolism of moso bamboo shoots during storage.

Structural polysaccharides are important components of plant cell walls, which are synthesized by glycosyltransferases like GT43. In this study, overexpression of PH02Gene04386 encoding glycosyltransferase 43 promoted the elongation of OE *Arabidopsis* seedling roots on the medium with UDP xylose as a carbon source (Figure 7A), suggesting PH02Gene04386 could increase the efficiency of xylose utilization. The change of xylan content and its distribution in PH02Gene04386 OE *Arabidopsis* in this study (Figures 7D,E) supported the important role of GT43 family members involved in xylan biosynthesis. Similar results were also found in *GhGT43Al* and *GhGT43C1* OE cotton plants (Li et al., 2014), *IRX9* and *IRX14* in *Arabidopsis*, rice and cotton (Wu et al., 2010), and *GT43A* and *GT43B* in polar (Ratke et al., 2018). A model to elucidate the regulatory mechanism involved in carbohydrate metabolism of moso bamboo shoots during storage was constructed (Figure 8). Overall, these results will not only be useful for understanding the physiological changes and molecular regulatory mechanisms of moso bamboo shoots during storage, but also provide an important potential candidate gene for the breeding related to xylan.

## Data availability statement

The datasets presented in this study can be found in online repositories. The names of the repository/repositories and accession number(s) can be found below: https://www.ncbi.nlm.nih.gov/, PRJNA840860 and https://www.ncbi.nlm.nih.gov/, PRJNA840882.

## Author contributions

ZL designed and performed the experiments and wrote the manuscript. XX constructed the conceptions and worked for the bamboo sample collection. KY and CZ assisted with Y1H assay. YL assisted in completing the histochemical staining. ZG checked the data analysis and revised the manuscript. All authors contributed to the article and approved the submitted version.

## Footnotes

1
https://www.omicshare.com/tools/Home/Soft/trend

2
www.biocloud.net

3
http://planttfdb.gao-lab.org/

4
https://www.zhaolab.org/psRNATarget/

## Supplementary material

Supplementary Figure 1 Gene expression analysis in moso bamboo during storage. **(A)** Correlation analysis between different samples based on gene expression. **(B)** Number of DEGs encoding transcript factors. **(C)** Number of DEGs between different comparison groups. S1, S2, S3, and S4 represent bamboo shoots stored for 0, 3, 6, and 12 d.

Supplementary Figure 2 GO **(A)** and KEGG **(B)** analyses of DEGs in cluster III of Rich Factor represents the value of enrichment factor which is the quotient of foreground value (the number of DEGs), and the larger the value, the more significant enrichment; Coloring indicates *P*-value with higher in red and lower in blue, and the lower *P*-value, the more significantly enriched. Point size indicates the number of DEGs.

Supplementary Figure 3 The expression patterns of DEGs involved in starch and sucrose metabolism. AMY, α-amylase; BMY, β-amylases; GBE, glucan branching enzyme; GH, glycosyl hydrolase; INV, fructosidase; glgA, starch synthase; glgC, glucose-1-phosphate adenylyltransferase; MGAM, maltase-glucoamylase; pgm, phosphoglucomutase; SPS, sucrose phosphate synthase; SUS, sucrose synthase; UGP, glucose-1-phosphate uridylyltransferase. The color bar indicates log_2_-based fragments per kilobase per million (FPKM) with higher in red and lower in blue.

Supplementary Figure 4 The expression patterns of DEGs involved in glycolysis. ALDO, fructose-bisphosphate aldolase; ENO, enolase; GAPDH, glyceraldehyde 3-phosphate dehydrogenase; gpmI. phosphoglycerate mutase; HK, hexokinase; PFK, phosphofructokinase; PGK, phosphoglycerate kinase; PK, pyruvate kinase. The color bar indicates log_2_-based fragments per kilobase per million (FPKM) with higher in red and lower in blue.

Supplementary Figure 5 Length and family classification of miRNA. **(A)** Length of known miRNA. **(B)** Length of novel miRNA. **(C)** Percentage of miRNA members in each family.

Supplementary Figure 6 Function analysis of miRNA-target pairs.

Supplementary Figure 7 WGCNA modules based on DEG data. **(A)** Cluster dendrogram. **(B)** Module–trait relationships. Each column corresponds to a flavor compound. The color of each cell at the row-column intersection indicates the correlation coefficient between the module and the substance. **(C)** Expression heat map and expression level of DEGs in the module.

Supplementary Figure 8 Validation of PH02Gene04386 overexpressed plants. **(A)** Overexpression vector of PH02Gene04386. **(B)** Screening of transgenic plants by PCR. **(C)** Detection of PH02Gene04386 expression in transgenic plants by qPCR. WT: Wild type *Arabidopsis*. L1, L3, L6, L7, L9: Transgenic lines. Lowercase letters a and b indicate significant differences (*P* < 0.05).
