# Supplementary figures and images for: Multifaceted analyses reveal carbohydrate metabolism mainly affecting the quality of postharvest bamboo shoots
Source: Front Plant Sci. 2022 Sep 21;13:1021161. doi: 10.3389/fpls.2022.1021161 (PMC9535365; doi:10.3389/fpls.2022.1021161)

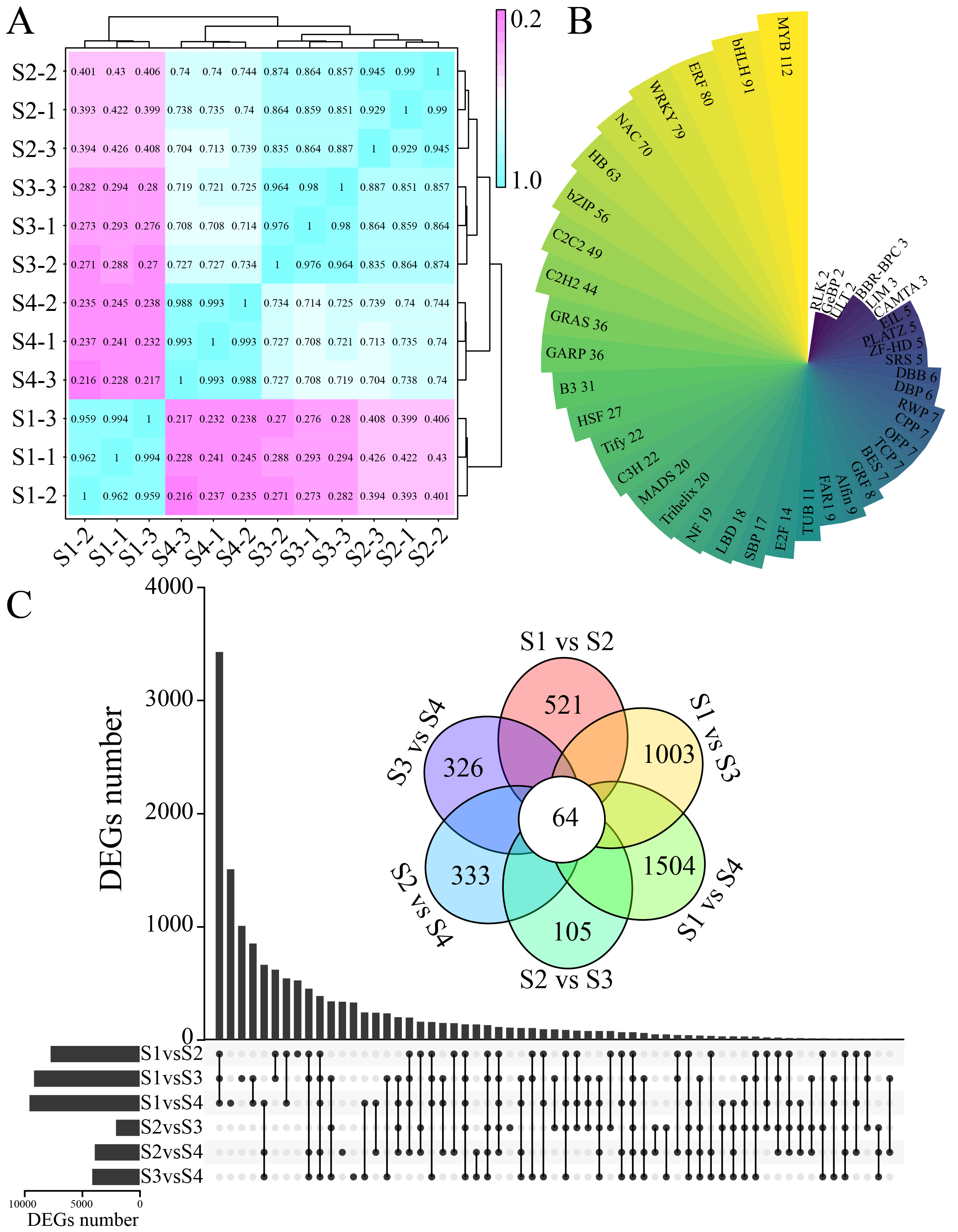

Supplement: Supplementary Figure 1 — Gene expression analysis in moso bamboo during storage. (A) Correlation analysis between different samples based on gene expression. (B) Number of DEGs encoding transcript factors. (C) Number of DEGs between different comparison groups. S1, S2, S3, and S4 represent bamboo shoots stored for 0, 3, 6, and 12 d. [file Image_1.JPEG]

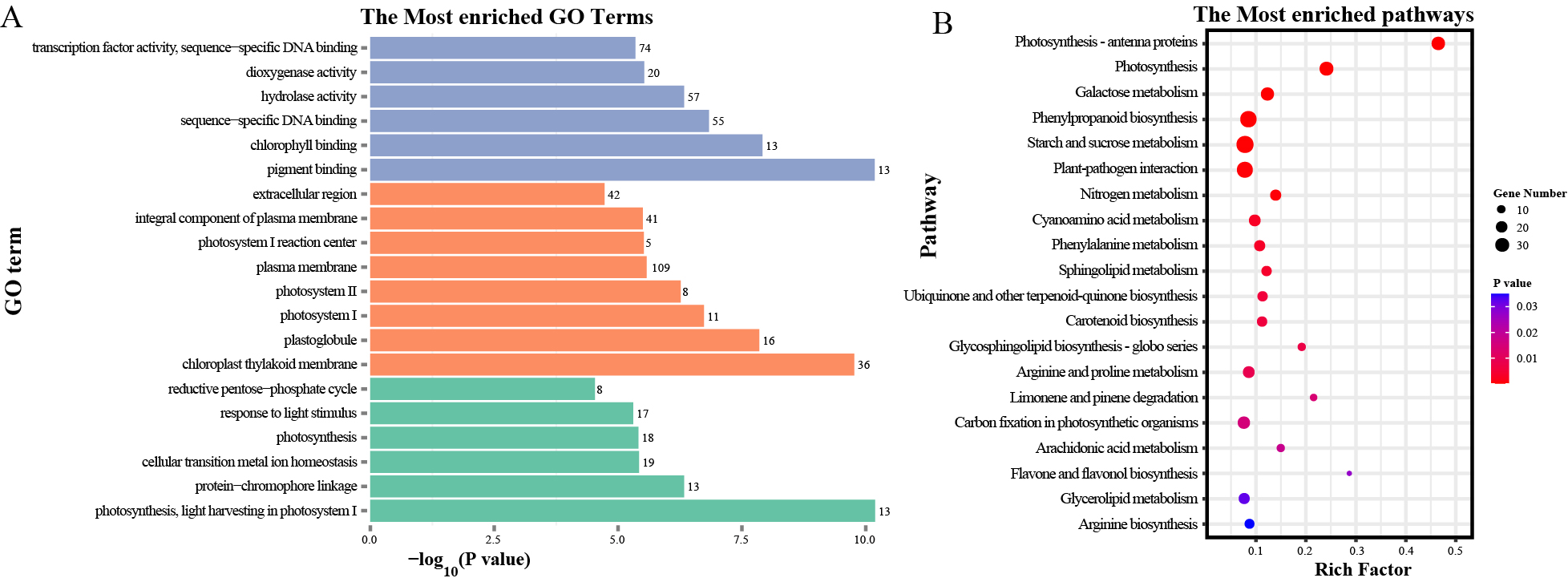

Supplement: Supplementary Figure 2 — GO (A) and KEGG (B) analyses of DEGs in cluster III of Rich Factor represents the value of enrichment factor which is the quotient of foreground value (the number of DEGs), and the larger the value, the more significant enrichment; Coloring indicates P-value with higher in red and lower in blue, and the lower P-value, the more significantly enriched. Point size indicates the number of DEGs. [file Image_2.JPEG]

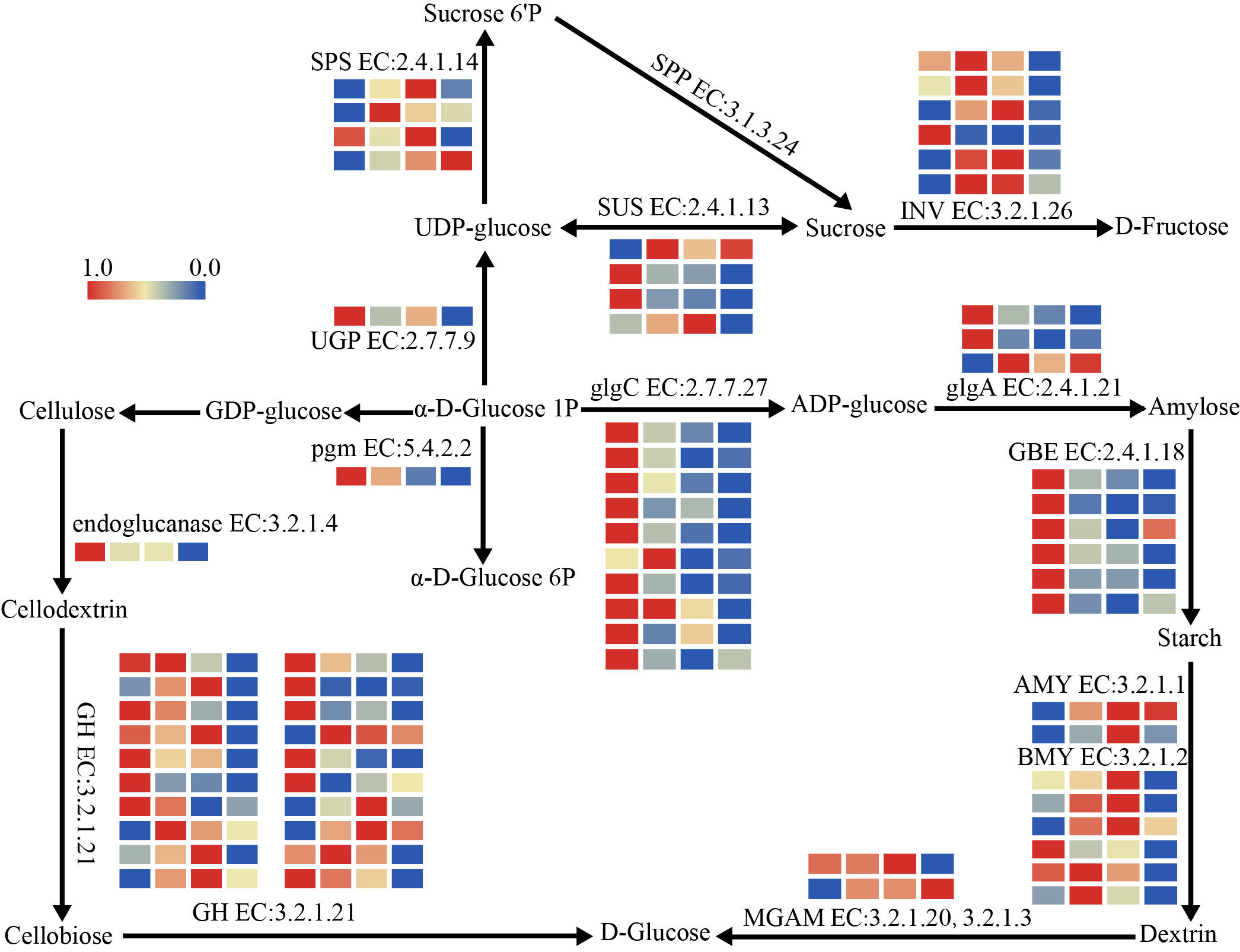

Supplement: Supplementary Figure 3 — The expression patterns of DEGs involved in starch and sucrose metabolism. AMY, α-amylase; BMY, β-amylases; GBE, glucan branching enzyme; GH, glycosyl hydrolase; INV, fructosidase; glgA, starch synthase; glgC, glucose-1-phosphate adenylyltransferase; MGAM, maltase-glucoamylase; pgm, phosphoglucomutase; SPS, sucrose phosphate synthase; SUS, sucrose synthase; UGP, glucose-1-phosphate uridylyltransferase. The color bar indicates log2-based fragments per kilobase per million (FPKM) with higher in red and lower in blue. [file Image_3.JPEG]

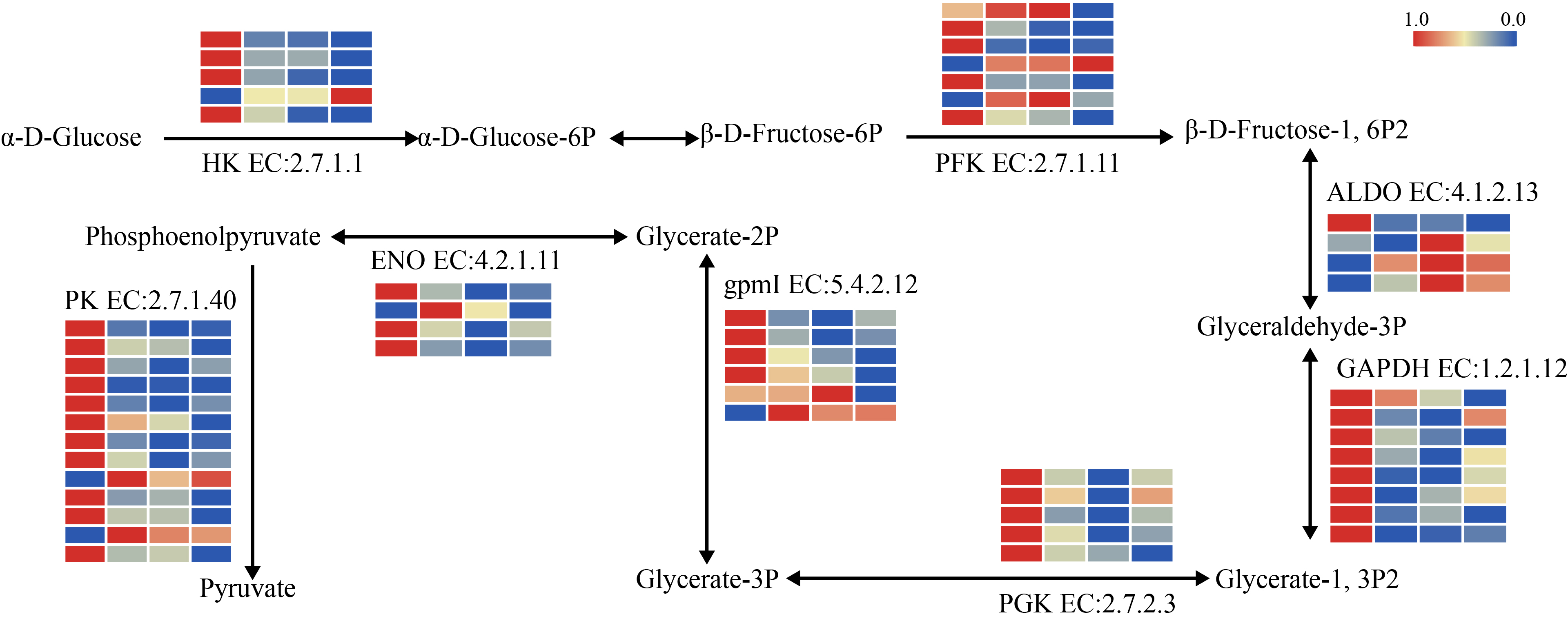

Supplement: Supplementary Figure 4 — The expression patterns of DEGs involved in glycolysis. ALDO, fructose-bisphosphate aldolase; ENO, enolase; GAPDH, glyceraldehyde 3-phosphate dehydrogenase; gpmI. phosphoglycerate mutase; HK, hexokinase; PFK, phosphofructokinase; PGK, phosphoglycerate kinase; PK, pyruvate kinase. The color bar indicates log2-based fragments per kilobase per million (FPKM) with higher in red and lower in blue. [file Image_4.JPEG]

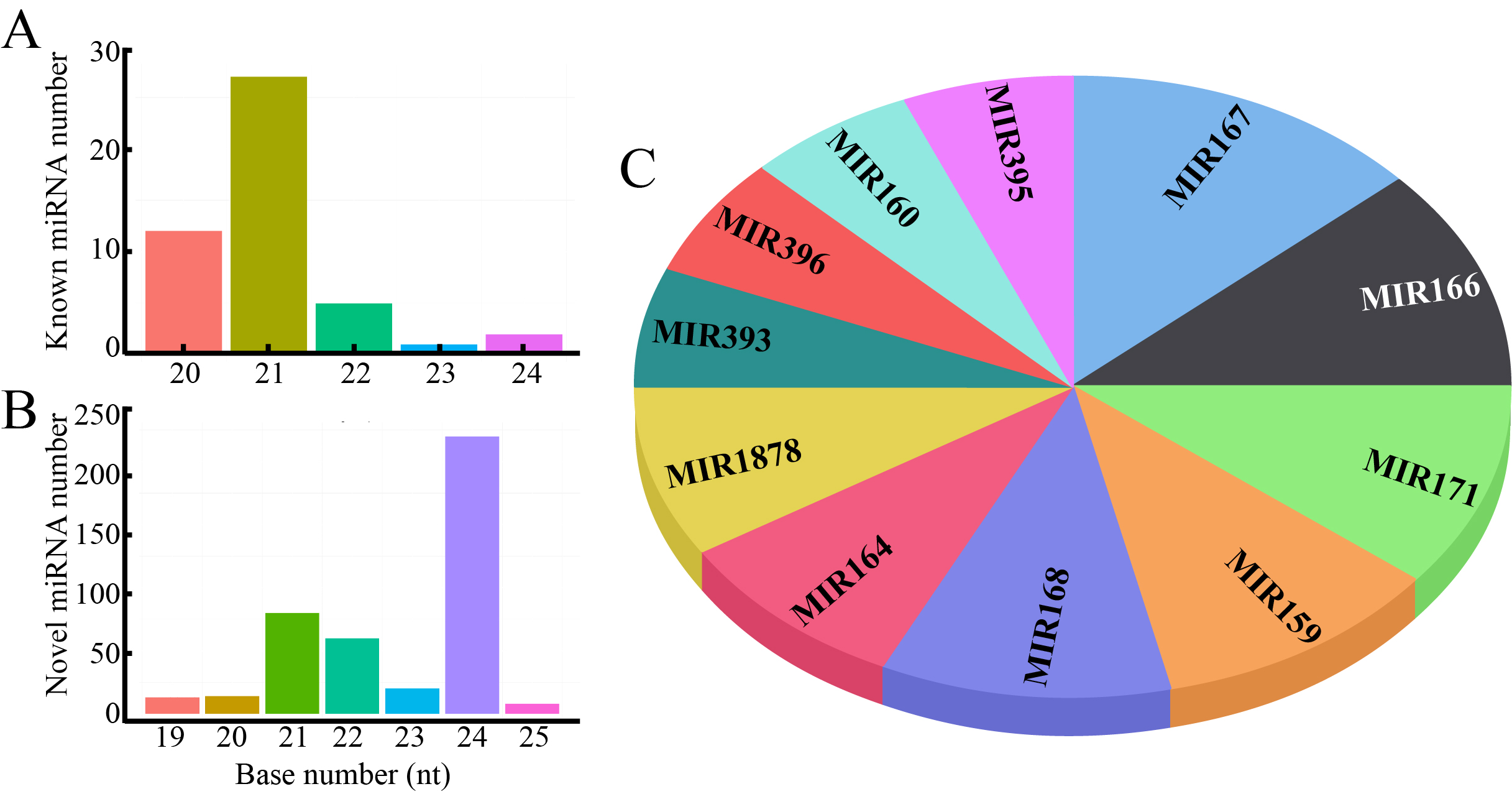

Supplement: Supplementary Figure 5 — Length and family classification of miRNA. (A) Length of known miRNA. (B) Length of novel miRNA. (C) Percentage of miRNA members in each family. [file Image_5.JPEG]

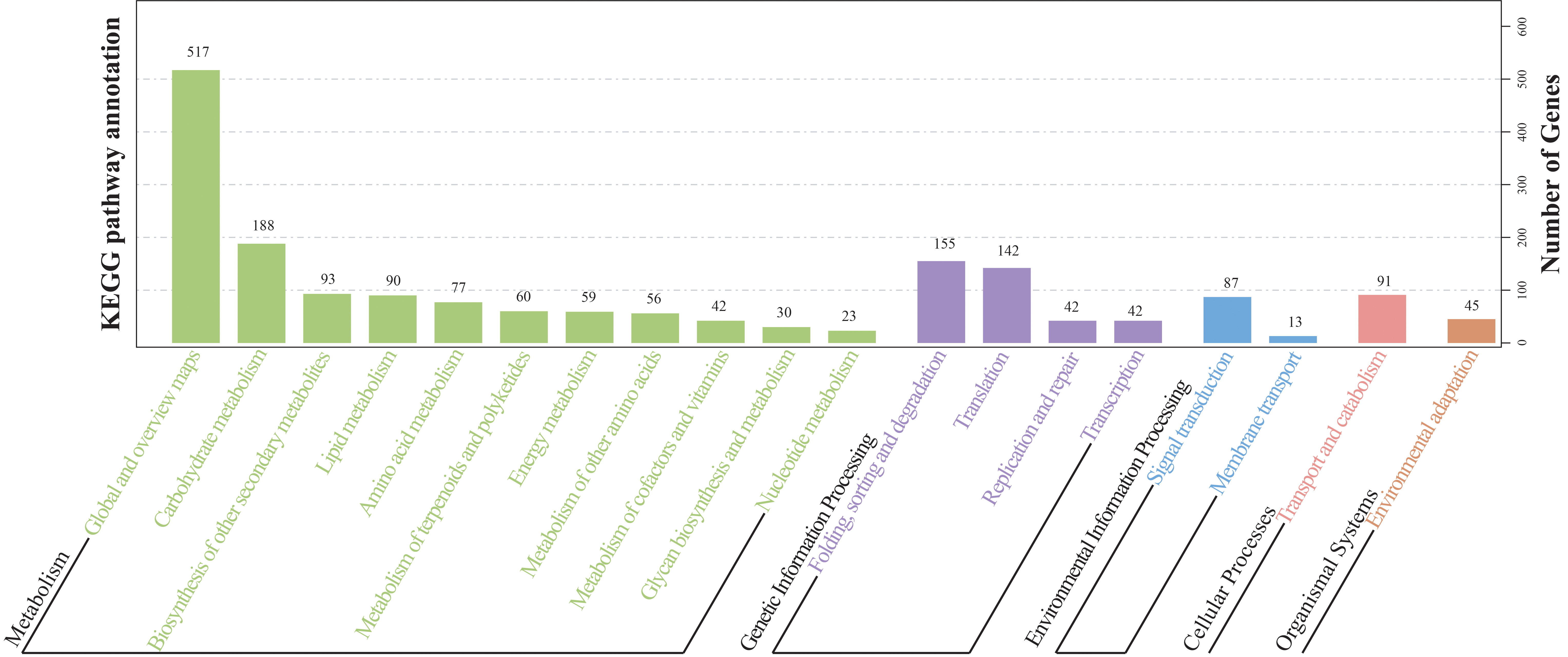

Supplement: Supplementary Figure 6 — Function analysis of miRNA-target pairs. [file Image_6.JPEG]

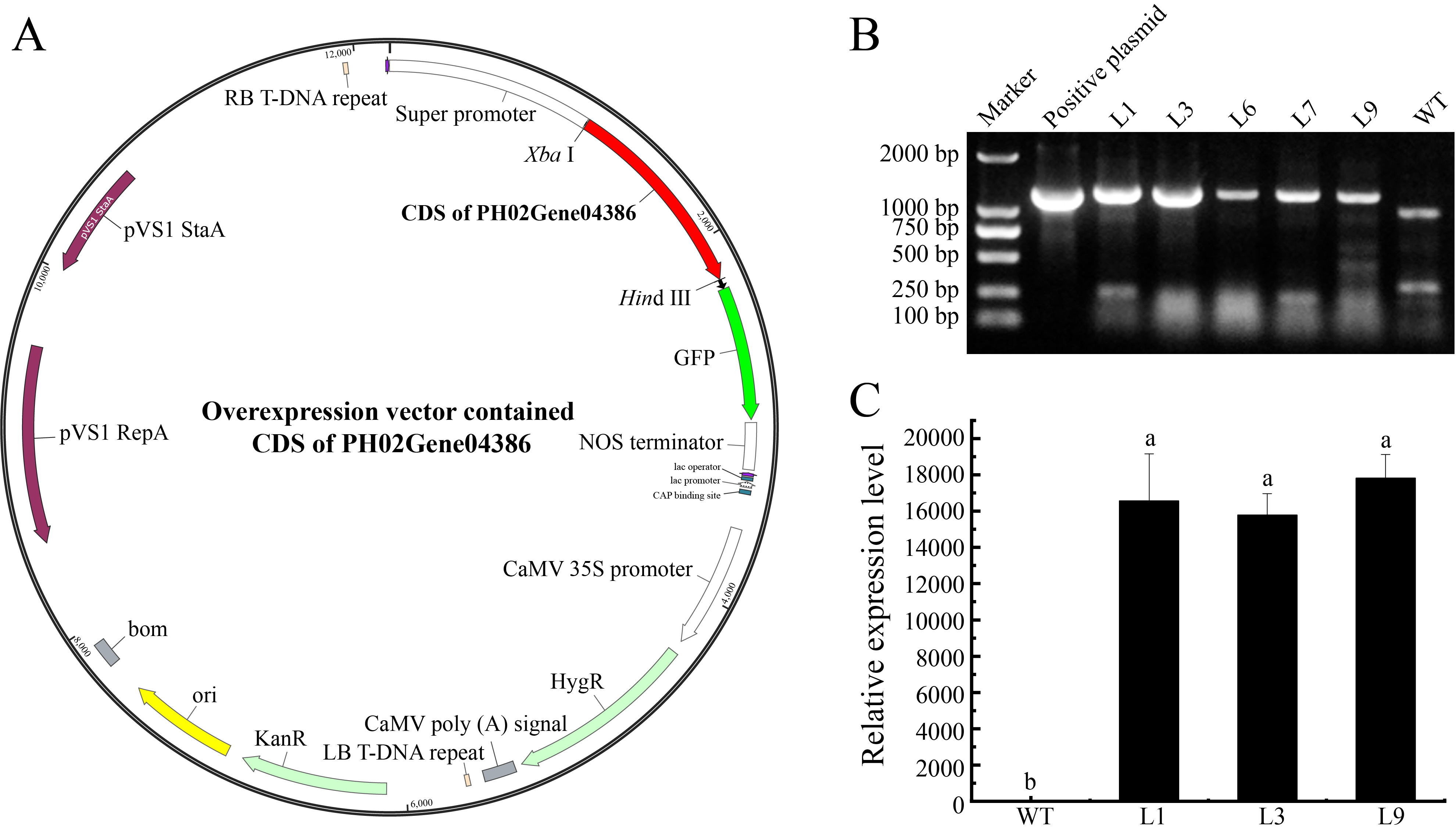

Supplement: Supplementary Figure 8 — Validation of PH02Gene04386 overexpressed plants. (A) Overexpression vector of PH02Gene04386. (B) Screening of transgenic plants by PCR. (C) Detection of PH02Gene04386 expression in transgenic plants by qPCR. WT: Wild type Arabidopsis. L1, L3, L6, L7, L9: Transgenic lines. Lowercase letters a and b indicate significant differences (P < 0.05). [file Image_8.JPEG]
